# Test-Retest Reliability and Minimum Difference Values of a Novel and Portable Upright Row Strength Assessment in Probation Officers

**DOI:** 10.3390/ijerph20032236

**Published:** 2023-01-27

**Authors:** Nicholas A. Buoncristiani, Jacob A. Mota, Gena R. Gerstner, Hayden K. Giuliani-Dewig, Eric D. Ryan

**Affiliations:** 1 Neuromuscular Assessment Laboratory, Department of Exercise and Sport Science, University of North Carolina at Chapel Hill, Chapel Hill, NC 27599, USA; 2 Carolina Center for Healthy Work Design and Worker Well-Being, University of North Carolina at Chapel Hill, Chapel Hill, NC 27599, USA; 3 Human Movement Science Curriculum, University of North Carolina at Chapel Hill, Chapel Hill, NC 27599, USA; 4 Department of Kinesiology and Sport Management, Texas Tech University, Lubbock, TX 79430, USA; 5 Human Performance Innovation Center, Rockefeller Neuroscience Institute, West Virginia University, Morgantown, WV 26505, USA

**Keywords:** isometric peak force, upper body strength, tactical, law enforcement officers

## Abstract

Upper body (UB) strength is important for occupational tasks and injury prevention in law enforcement officers (LEOs). Portable, reliable, and cost-effective assessments are needed to examine UB strength among LEOs in field settings. The purpose of this study was to examine the test-retest reliability and minimum difference (MD) values of a novel and portable isometric upright row assessment in probation officers. Thirty certified probation officers (18 women; age = 38.9 ± 9.0 years, body mass = 98.8 ± 27.1 kg, stature = 171.4 ± 14.0 cm) volunteered for this investigation. Testing occurred on-site across two sessions (2–5 days apart). Participants stood upon an aluminum plate with a chain attached to a handle and dynamometer. They grasped the handle with a pronated grip, two cm below the umbilicus, and performed three isometric maximal voluntary contractions. Intraclass correlation coefficient (ICC2,1), standard error of the measurement (SEM), and MD values were calculated. Results indicated no significant systematic error (*p* = 0.080) across sessions. The ICC2,1, SEM, and MD values for UB strength were 0.984, 27.20 N (4.1% of the mean), and 75.38 N (11.3% of the mean), respectively. These data suggest this isometric upright row assessment is a reliable, portable, and cost-effective measure of UB strength to assess and monitor LEOs in field settings.

## 1. Introduction

Law enforcement officers (LEOs) are essential public safety personnel, with occupational tasks that include high physical and psychological demands [1,2]. As of 2020, there were nearly 800,000 LEOs employed in the United States, with an expected increase of nearly 10 percent by 2030 [3]. The unpredictable nature of this occupation requires sudden transitions from sedentary activity (i.e., driving, paperwork), to tasks that require high-physical demand (i.e., foot pursuit and suspect apprehension) [2,4]. Due to the high physiological stress and hazardous nature of this occupation, LEOs have an injury rate of nearly 500 per 10,000 workers, which is over four times the national average (107.1 per 10,000), resulting in a large financial burden [5,6]. 

Previous investigations have noted that upper body (UB) strength is an important factor impacting occupational performance and injury risk [7,8]. For example, the primary critical job tasks for LEOs include maneuvering over fences, subduing and controlling suspects, and transporting victims or offenders, all of which require some degree of UB strength [1]. Previous studies have found low isometric strength to be associated with poorer performance during simulated occupational tasks and shooting accuracy in LEOs and recruits [7,8]. In addition, low isometric UB strength (i.e., grip strength) has also been shown to be associated with a greater injury risk in LEO recruits [8]. 

The most common laboratory assessment of UB strength is typically performed using isokinetic dynamometry [9,10]. However, due to its cost and lack of portability, investigators have utilized handheld dynamometry (HHD) to assess muscular strength in field and clinical settings [9,10]. Previous studies [11,12] have noted a lack of standardization of participant placement and evaluator positioning, and dependence on evaluator strength as key HDD weaknesses that may impact its consistency, especially when examining larger muscle groups. Given the importance of UB strength in LEOs, future studies are needed to identify reliable, portable, and cost-effective UB multi-joint strength assessments. Further, the determination of minimum difference (MD) values may be important for researchers and LEO administrators to determine if a difference or change in UB strength can be considered real [13]. Thus, the purpose of this study is to examine the test-retest reliability and MD values of a novel and portable isometric upright row assessment in probation officers, a subset of LEOs. We chose to use a validated, commercially available dynamometer [14] during a common bilateral, easy-to-perform UB exercise, often prescribed in resistance exercise programs [15].

## 2. Materials and Methods

### 2.1. Participants

Thirty certified probation officers (18 women; all demographic data are in Table 1) volunteered for this investigation. Before testing, all participants completed and signed an approved consent form, and completed a health history questionnaire. None of the participants reported any current neuromuscular or metabolic diseases, or musculoskeletal injuries sustained within the previous three months that would preclude them from participating in the testing. In addition, none of the participants were involved in an active workers’ compensation or personal injury case, or were trying to become pregnant. This study was approved by the University’s Institutional Review Board (#16–3295) and all participants were informed of the benefits and risks of the investigation prior to signing an institutionally-approved informed consent document to participate in this study. 

### 2.2. Experimental Design

This study used a repeated-measures design to examine the test-retest reliability of a novel UB isometric assessment among probation officers. Testing occurred across two separate sessions that were scheduled 2–5 days apart and were completed at the same time of the day (± three hours). All testing occurred on-site at local probation-officer offices in North Carolina.

### 2.3. Isometric Strength Testing

**Figure 1:**
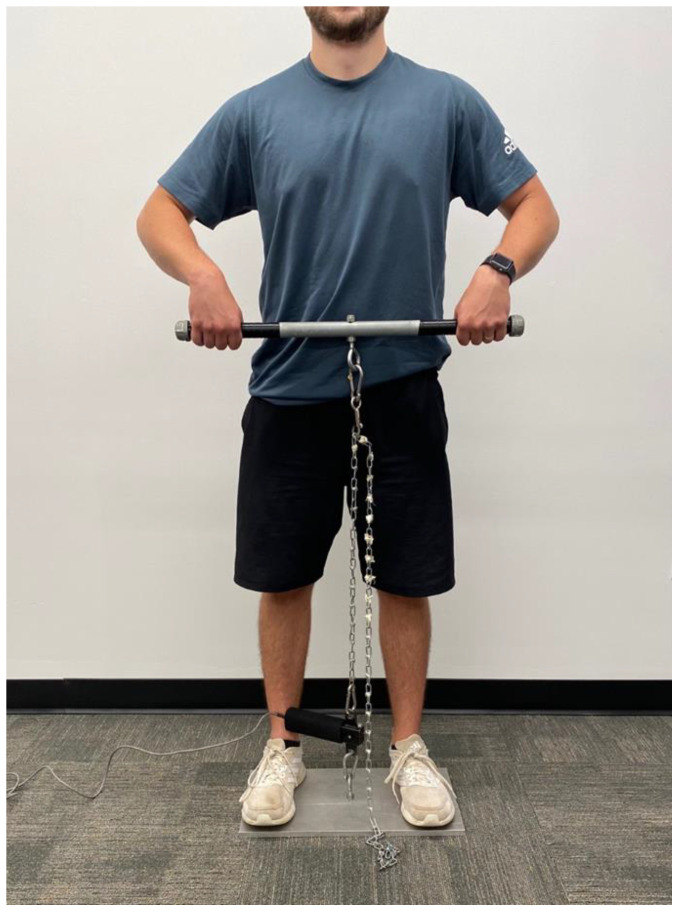
Example of the upper body isometric upright row assessment. The handle is placed approximately two centimeters below the umbilicus.

Upper-body isometric strength testing occurred on a custom-built, portable, aluminum plate (which the participants stood on), that included a tension dynamometer (TSD121C, BIOPAC Systems Inc., Goleta, CA) attached in series with a metal chain and handle (Figure 1). Participants grasped the handle with a pronated grip, approximately shoulder-width apart. The length of the chain was adjusted so that the handle was approximately two cm below the umbilicus for each participant and kept the same for each testing session. Following a brief warm-up consisting of three submaximal isometric contractions (50–75% of perceived maximal exertion) lasting four seconds, participants then performed three isometric maximal voluntary contractions (MVC), with a 2-minute rest period between attempts. Participants were initially instructed to pull the bar sub-maximally to remove the slack in the chain immediately prior to the start of the MVC, as determined with visual feedback of force production. Once the baseline force was completely steady, participants were given strong verbal encouragement to pull as hard and as fast as possible during each contraction for 3–4 s.

### 2.4. Signal Processing

Force (N) was sampled at 2KHz with a Biopac data acquisition system (MP150WSW, Biopac Systems Inc., Goleta, CA, USA) and stored on a personal computer (ThinkPad T420; Lenovo, Morrisville, NC, USA). Custom-written software (LabVIEW 17; National Instruments, Austin, TX, USA) was used to process all the signals offline. Signals were filtered with a zero-phase shift, fourth order, low-pass Butterworth filter (150 Hz) [16]. Peak force for the MVCs was defined as the highest 500 ms epoch during the plateau of the MVC.

### 2.5. Statistical Analysis

Test-retest reliability for UB isometric strength was evaluated using the procedures described by Weir [13] and analyzed using a custom-written software program (Microsoft Excel, Microsoft, Redmond, WA, USA). A one-way repeated-measures analyses of variance (ANOVAs) were used to examine the systematic variability across testing days. The intraclass correlation coefficient (ICC) was calculated from model ‘2,1’, as described by Shrout and Fleiss [17], because it has been suggested that the ICCs generated with this model can be generalized to other research laboratories conducting field-based assessments. Further, the standard error of measurement (SEM) and MD values were calculated based on the recommendations of Weir [13], using the mean square error term from the ANOVA, and expressed as a percentage of the mean. Alpha levels were set a priori at 0.05 to determine statistical significance.

## 3. Results

Our participant demographics are similar to and representative of probation officers in the state of North Carolina [18]. There was no significant systematic error (*p* = 0.080) between day 1 (658.50 ± 228.14 N) and day 2 (671.25 ± 217.70 N) UB strength values. The ICC2,1, SEM, and MD values for UB strength were 0.984, 27.20 N (4.1% of the mean), and 75.38 N (11.3% of the mean), respectively.

## 4. Discussion

The findings of the present study indicated no significant systematic error across testing sessions and acceptable relative (ICC2,1 = 0.984) and absolute (SEM = 27.2 N, SEM% = 4.1%) consistency values. To the authors’ knowledge, this is the first study to assess the test-retest reliability of a portable UB isometric upright row strength assessment in first responders. Previous papers have examined the test-retest reliability of various portable UB assessments, ranging from “make” or “break” HHD, to fixed isometric assessments [9,10,11]. Of these, previous work utilizing isometric assessments to examine UB strength have reported similar test-retest reliability. For example, Holt et al. [9] examined the reliability of portable isometric shoulder strength assessments, which resulted in similar relative (ICC = 0.91–0.96) and absolute (SEM = 2.21–3.87 Nm, SEM% = 7.13–9.59%) values when compared to our novel strength assessment. 

From a practitioner perspective, the MD values from this study suggest a change of at least 75.38 N (11.3% of the mean) is needed to be considered a real change in UB isometric upright row strength as a result of an intervention, treatment, or condition, which is similar to the findings reported by Holt et al. [9]. Previous authors [19,20,21] have also examined sex-related differences, training interventions (pre- and post-resistance training), and upper-body injury (participants recovering from radial fracture versus uninjured participants) on UB strength reporting differences of 216 N, 76 N, and 118 N, respectively. Based on the results of our investigation and previous studies, this assessment can reliably detect changes in UB strength seen in common clinical and training scenarios. 

It is important to discuss our limitations. The current assessment examined UB isometric strength during an upright row. It is unclear if this setup can be used to examine other specific measures of UB strength (e.g., shoulder flexion, extension) traditionally examined with isokinetic dynamometry. 

## 5. Conclusion and Practical Applications

Previous studies [7,8] have noted that UB muscular strength is critically important for various job-specific occupational tasks and injury prevention. For example, decreased UB strength is associated with failing simulated occupational assessments, poor marksmanship, and injury status in LEOs [8]. For tactical strength and conditioning researchers and practitioners, it is often more feasible to assess UB muscle strength in LEOs at their local offices. Common portable tests that employ HHD are often dependent on the clinician’s strength and experience level, while lacking testing standardization that may impact the usefulness of HHD to assess UB strength [11,12]. Future UB assessments using validated, commercially available equipment are needed to overcome these limitations. The findings from this study demonstrated that the UB isometric upright row is a reliable multi-joint UB muscle strength assessment that is portable, cost-effective, easy to implement, and sensitive to changes commonly seen in many clinical and training situations.

## Figures and Tables

**Table 1:** Mean ± standard deviation (SD) and range values for the demographic variables.

	Mean ± SD	Range
Age (yrs)	38.9 ± 9.0	25.0–55.0
Stature (cm)	171.4 ± 14.0	154.5–199.1
Body Mass (kg)	98.8 ± 27.1	52.0–185.4
Body Mass Index (kg/m^2^)	33.2 ± 5.8	21.1–47.2

## Data Availability

Deidentified individual data that supports the results will be shared upon a reasonable request beginning 12–24 months following publication, provided the investigator who proposes to use the data has approval from an Institutional Review Board (IRB), Independent Ethics Committee (IEC), or Research Ethics Board (REB), as applicable, and executes a data use/sharing agreement and data management plan with UNC.
